# Risk of Repeated Adverse Effects following Booster Dose of mRNA COVID-19 Vaccine: Results from the MOSAICO Study

**DOI:** 10.3390/vaccines11020247

**Published:** 2023-01-22

**Authors:** Pietro Ferrara, Domenico Ponticelli, Lorenzo Losa, Claudia Romeo, Roberto Magliuolo, Andrea Vitale, Anna Zampella, Lucia Alleanza, Mario Borrelli, Beniamino Schiavone, Lorenzo Giovanni Mantovani

**Affiliations:** 1 Center for Public Health Research, University of Milan–Bicocca, 20900 Monza, Italy; 2 Istituto Auxologico Italiano—IRCCS, 20165 Milan, Italy; 3 Pineta Grande Hospital, 81030 Castel Volturno, Italy

**Keywords:** adverse effects following immunization, AEFI, mRNA COVID-19 vaccine, healthcare workers, SARS-CoV-2, safety

## Abstract

The successful deployment of safe and effective vaccines against coronavirus disease 2019 (COVID-19) has been crucial in reducing the global disease burden. Owing to the need for vaccination series over time, continuous observational studies are needed to estimate the COVID-19 vaccine response in real-world conditions. In particular, the detection, assessment, and understanding of adverse effects following immunization (AEFI) with a COVID-19 vaccine are crucial to better address vaccination strategies. Therefore, this study aimed to investigate the risk of repeated AEFI post-administration of a booster dose of mRNA COVID-19 vaccine in a sample of healthcare workers (HCWs) in an Italian teaching hospital. The data on any local and systemic AEFI were studied in multivariate Poisson regression analyses to model the association between the incidence of each postvaccination symptom and its prior reporting after the administration of the previous doses. Overall, compared with the primary vaccination series, the majority of post-third dose AEFI were less reported. The results from multivariable models showed that the likelihood of reporting an AEFI after the third dose was higher in those who experienced the same postvaccination symptom after the second dose (all AEFI except for itch at injection site) and, although not significant for all AEFI, after the first dose. Any associations with age, gender, smoking habits, previous SARS-CoV-2 infection and other characteristics, as well as the health impact of AEFI were also assessed. Taken together, the results from this research support reframe AEFI symptoms as signals of a robust postvaccination reaction as well as of common vaccine response, and they add important data to inform booster vaccination strategies in HCWs and, extensively, in the adult population.

## 1. Introduction

Extensive evidence from real-world data supports the safety and effectiveness of vaccines against coronavirus disease 2019 (COVID-19), protecting against serious illness, hospitalization and death from COVID-19 [1,2,3].

The constantly evolving epidemiology of severe acute respiratory syndrome coronavirus 2 (SARS-CoV-2) [4], the diffusion of new viral variants (more recently, the B.1.1.529 Variant of Concern (named Omicron) and descendent lineages [5]), and the waning of protection offered by vaccination over time [6,7] has impacted the SARS-CoV-2 transmissibility, the occurrence of breakthrough infections and the COVID-19 vaccine effectiveness, with the need for boosting immunity to ensure continuity of protection [8].

The safety profile of COVID-19 vaccines is under continuous evaluation, with the vast majority of known adverse effects following immunization (AEFI) being minor and self-limiting [9,10,11]. Indeed, the investigation of adverse effects following immunization (AEFI) is key to monitoring the safety of vaccination series and acceptance of repeated doses in general and in specific populations [12,13,14]. Understanding AEFI predictors after COVID-19 vaccines gives the opportunity to better design prevention strategies and encourage trust building in vaccination campaigns against COVID-19 [2,14,15].

While growing research has compared AEFI onset and severity after primary vaccination and booster series, these studies fostered more in-depth safety monitoring of COVID-19 vaccines, also due to the lack of experience with massive vaccination programs with novel mRNA platforms [14,15,16,17,18,19]. Yet, to our knowledge, only limited evidence has been published on the effect of having previously reported a postvaccination symptom on the incidence of post-third dose AEFI [20]. Therefore, to fill this literature gap, this study aimed at the investigation of the adverse events following a booster dose of the licensed mRNA COVID-19 vaccines, comparing the incidence of these AEFI between doses and understanding possible predictors of their onset. In particular, the main research goal is to estimate the risk of repeated adverse effects following a booster dose of the mRNA COVID-19 vaccine in a sample of HCWs enrolled in the MOSAICO study (“Monitoraggio di Breakthrough infectionS dopo dose Addizionale di vaccino antI-COVID-19 a mRNA in operatori sanitari”), which defines a longitudinal observational study designed to investigate the safety and effectiveness of booster immunization with the mRNA COVID-19 vaccine in the HCWs of the Pineta Grande Hospital (Castel Volturno, Caserta, Italy) [8].

## 2. Materials and Methods

### 2.1. Study Design and Population

The aims of this study included the description of post-third dose AEFI and the study of the association between their incidence and the previous experience of the same symptoms after primary vaccination, also considering a set of individual participants’ characteristics.

This research, built as a retrospective chart review, is part of the MOSAICO study, complete methods of which have been presented elsewhere [8]. Briefly, all hospital workers—HCWs and non-HCWs (the latter being all hospital workers who were not in close contact with patients)—who received a booster dose of an mRNA vaccine (BNT162b2 or mRNA-1273) between 2 October 2021 and 15 February 2022 were asked to take part in the study. Participation was voluntary, and vaccinees were not offered any incentive and were informed about their right to withdraw at any time without penalty.

Participants who signed provided the informed consent were interviewed about their demographics and professional characteristics—including gender, age, professional role, smoking habits, health status (i.e., autoimmune disease, immunosuppressive therapy (no = 0; yes = 1); type of booster vaccine (BNT162b2 = 0; mRNA-1273 = 1); previous laboratory-confirmed SARS-CoV-2 infection; AEFI occurrence after booster dose; and the health impact of adverse events following the booster dose (as a proxy of their severity).

Data on any local (pain, redness, swelling, and itch at injection site) and systemic AEFI (tiredness and fantigue, headache, muscle pain, joint pain, chills, fever, swelling of lymph node, abdominal pain, nausea, vomiting, diarrhea, breathing problems, and swelling of the face, tongue, throat, dizziness, and other uncatalogued AEFI which may be experienced) that occurred within seven days after the administration of each vaccine dose were registered. The list of included AEFI aligns with the previous research on COVID-19 vaccine safety [14,20].

### 2.2. Statistical Analysis

A descriptive analysis was carried out to describe cohort characteristics and outcomes. Continuous variables were summarized as median and interquartile range (IQR), based on their distribution. Categorical variables were expressed as absolute and relative frequency.

Multivariate Poisson regression analyses with robust standard errors were conducted to model the incidence of each post-third dose AEFI. This enabled relative risks (as incidence rate ratios, RR) and 95% confidence intervals (95%CI) associated with a set of explanatory variables, including having experienced the same AEFI after first dose (no = 0; yes = 1); having experienced the same AEFI after second dose (no = 0; yes = 1); gender (men = 0; women = 1), age (continuous, in years), professional role (non-HCW = 0; HCW = 1), and smoking habits (never/former smoker = 0; current smoker = 1); previous SARS-CoV-2 infection (no = 0; yes = 1); autoimmune disease (no = 0; yes = 1); immunosuppressive therapy (no = 0; yes = 1); type of booster vaccine (BNT162b2 = 0; mRNA-1273 = 1); and time interval between primary vaccination cycle and booster dose (continuous, in weeks). A stepwise backward selection procedure was followed by setting a significance level of <0.4 as criterion for variables to enter the final multivariate Poisson model. To ensure consistency of the analyses, multivariate models were fitted for those AEFI that showed a cumulative incidence greater than 5% in the whole sample.

Subsequently, we fitted multinomial logistic regression models to investigate the health impact (i.e., inability to perform normal daily activities, inability to work and need of medical care) of experiencing at least one post-third dose AEFI and possible relevant predictors (gender, age, professional role, and smoking habits; previous SARS-CoV-2 infection; autoimmune disease; immunosuppressive therapy; type of booster vaccine), identified through a stepwise regression strategy (*p*-value equal to or greater than 0.4 for exit). The following models were constructed: odds of inability to perform normal daily activities due to AEFI (Model 1); odds of inability to work due to AEFI (Model 2); odds of seeking medical care due to AEFI (Model 3). In the logistic regression models, adjusted odds ratios (ORs) and 95% CIs expressed the effect estimates.

All *p*-values were two-sided and ≤0.05 assumed as statistically significant. Analyses were conducted with statistical software STATA version 17 (StataCorp. 2021, College Station, TX, USA) [21].

## 3. Results

The whole MOSAICO cohort consisted of 320 participants: ten individuals with a laboratory-confirmed SARS-CoV-2 infection within 6 months after the second dose were not administered the booster, as per Italian Ministry of Health regulations. Fifty participants (16.1%) were previously infected with SARS-CoV-2, while nine (3.0%) did not receive the second vaccine dose due to an infection occurring after the first dose or having received one-dose vaccines. The complete flowchart of cohort creation is presented in a previous MOSAICO publication [8]

Overall, a total of 310 recipients received an mRNA COVID-19 vaccine for boosting immunity after a median time of 36 weeks (IQR 35–42) since the primary vaccination cycle, with 83.5% having received the BNT162b2 vaccine. Of the whole sample, 258 (83.2%) individuals reported at least one AEFI. The baseline characteristics of the study population are presented in Table 1. 

The cumulative incidence of AEFI after the three doses of the COVID-19 vaccination series is detailed in the Appendix (Table A1): compared with the previous administration, the majority of post-third dose AEFI was less reported, with the exception of swelling of lymph nodes (+4.5% than second dose), abdominal pain (+1.6%), and shortness of breath (+0.7%).

Table 2 summarizes the results from multivariate Poisson regression analyses in terms of adjusted RRs and 95% CIs. These indicated that the probability of self-reporting one of all included AEFI (except for itch at injection site) was higher in those who experienced the same AEFI after the second dose, with RRs ranging from 3.56 (95% CI, 2.33–5.44) for fatigue and tiredness to 16.68 (95% CI, 4.96–56.06) for nausea. As regards the association with the reporting of the same AEFI after the first inoculation, significance was reached only for pain at the infection site (RR 1.90; 95% CI, 1.16–3.09), itch at the infection site (RR 9.95; 95% CI, 2.01–49.42), fatigue and tiredness (RR 1.56; 95% CI, 1.12–2.16), headache (RR 1.91, 95% CI, 1.31–2.80), joint pain (RR 1.54; 95% CI, 1.08–2.18), chills (RR 1.49, 95% CI, 1.06–2.09), fever (RR 1.51, 95% CI, 1.05–2.14), abdominal pain (RR 5.31, 95% CI, 1.31–21.47), and nausea (RR 2.65, 95% CI, 1.03–6.82). 

As a proxy of AEFI gravity, participants were also asked whether they were temporarily unable to perform normal daily activities and to work, as well as whether they sought medical care (Table 3). No severe AEFI requiring emergency department or hospital access were reported.

When estimating behaviors of recipients experienced at least one AEFI after third dose, gender, age, professional role, smoking habits, previous SARS-CoV-2 infection, autoimmune disease, immunosuppressive therapy, and type of booster vaccine were adjusted for in the multivariable logistic regression analyses (Table 4): odds of inability to perform normal daily and work activities were significantly higher in women and in those with autoimmune disease (Model 1 and 2), while being a HCW was associated with a lower value for odds of inability to work (Model 2). The odds of seeking medical care due to AEFI were significantly higher in mRNA-1273 vaccine recipients (Model 3).

## 4. Discussion

In this observational study, important results were yielded regarding the incidence of AEFI post-booster dose of the mRNA COVID-19 vaccine and the risk of repeated AEFI. Overall, four out of five booster recipients reported at least one AEFI after their booster dose, the more frequent of which were pain at injection site, tiredness and fatigue, muscle pain, and headache. A wide range of AEFI have been described among the participants, but all were previously described as common non-severe signals of postvaccination reactions of the immune system [14,16,20,22]. The only atypical reported side effects were reactivation of herpes zoster and left-sided facial numbness in BNT162b2 platform recipients. The first was seen firstly during the primary cycle in a 63-year-old woman with an autoimmune disease, and then in a 60-year-old immunocompetent man after the booster dose. Facial numbness developed in a 35-year-old immunocompetent man (after both first and second doses). Pharmacovigilance data described a low risk of temporary facial paralysis following COVID-19 vaccination, in line with other viral vaccines, and possible etiology may be related to the mRNA/lipids-induced activation of innate immunity and resultant interferon production [23,24,25]. Similarly, research found that reactivation of herpes zoster could follow the administration of different COVID-19 vaccines (with an estimated incidence of 7.1 (95% CI, 4.1–11.5) per 1,000,000 doses of BNT162b2), probably owing to transient immunomodulation following vaccination [26,27].

The most common post-booster AEFI were pain at injection site (73.6%), muscle pain (43.2%), headache (38.1%), and joint pain (36.1%), while less than one-third reported chills (29.0%) and fever (21.6%) (Appendix, Table A1). Overall, the vast majority of the investigated AEFI showed a lower reporting frequency compared with primary vaccination (apart from swelling of lymph nodes, abdominal pain, and shortness of breath). Of note, some AEFI amongst the most frequent ones—such as headache, muscle pain, joint pain, chills, and fever—were reported from a higher number of participants at second shot, dropping back with booster. This may intuitively be related to stronger postvaccination reaction with closer time intervals during the primary vaccination cycle, though time since primary cycle has no impact on future AEFI in our work.

Beyond the crude incidence of the studied symptoms, this research was designed with the main objective of predicting the risk of repeated AEFI—having experienced them after the first and second dose—according to other vaccinees’ characteristics such as age, gender, smoking habits, professional role, SARS-CoV-2 infection before booster vaccination, autoimmune comorbidities, concomitant immunosuppressive therapy, type of vaccine platform, and time since primary vaccination cycle.

Except for itch at injection site (and for AEFI excluded from the analysis for low incidence), having experienced each of the catalogued symptoms after the second dose highly predicted its occurrence as post-third dose symptom, with effect estimates that revealed a three-fold or greater risk for all AEFI, up to RR of 15.36 (95% CI, 8.97–26.30) for redness at injection site and 16.68 (95% CI, 4.96–56.06) for nausea. In this regard, it is also worth noting that the magnitude of estimated RRs is not associated with the cumulative incidence of AEFI, although the analyses modelled narrower confidence intervals for those more frequently reported.

The hallmark symptoms experienced after vaccination are common signals of immune system response and, thus, vaccine effectiveness. As regards systemic AEFI—such as fever, chills, muscle and joint pain—extensive literature reveals that these are part of an adequate immune reaction with the stimulation of both the innate and the adaptive arms of the immune system, and the release of cytokines and signaling pathways associated with immune cell function which are also responsible of thermogenesis and other systemic signs and symptoms [28]. Indeed, seroepidemiological surveys found that several side effects of mRNA vaccination—such as fever, chills, headache, fatigue, malaise, and nausea, as well as all AEFI analyzed together—accompanied vaccine-induced elicitation of antibody against SARS-CoV-2 spike protein (anti-S-RBD IgG) [29,30].

Consecutive boosters prompt the immune system against the waning of the correlate of protection after vaccination [7,30], which clearly explains the re-occurrence of AEFI, as found in this study. Here, we found that the risk of repeated AEFI could be predicted by individual predisposing factors (including age, gender, smoking status, health status, etc.) which have an underlying effect on immune response to vaccine.

The analyses did not find a correlation between the age of vaccinated HCWs and the risk of repeated AEFI, except for a slight increase in joint pain and chills with increasing age (2% per year). According to previous research, age is associated with lower anti-S-RBD (receptor binding domain) IgG after booster dose and with lower magnitude of memory B cell responses [30,31], as in the case of immune response after SARS-CoV-2 infection in vaccine-naïve individuals [32]. However, being limited to a working-age sample, our paper excluded upper age groups in which this effect may be seen.

We also noted that re-occurrence of tiredness/fatigue and headache was higher in women, likely owing to a more effective innate and adaptive immune response. Indeed, this finding mirrors differences in sex-disaggregated data on AEFI risk, with variation in hormones, genes, immune system activation, and pharmacokinetics proposed as possible explanations [14,20,33,34,35].

A recent research study by Herring et al. found that booster COVID-19 vaccine triggered a robust immune response, regardless of whether recipients had been previously infected with SARS-CoV-2 more than 3 months earlier, and found no difference in antibody levels in those who tested positive less than 3 months before [35]. The absence of difference in the immune activation in previously infected individuals agrees with our results showing that having tested positive did not significantly increase the risk of repeated AEFI. This may not apply to the arm irritation and pain after injection that showed a 17% (95%CI, 1–34) higher likelihood of occurrence. However, these symptoms are mediated by local immune reaction and the mechanism of muscle reactogenicity and irritation to both injection and other vaccine components [36].

The risks of repeated muscle pain, fever and nausea were significantly lower in tobacco smokers. The study of smoking in the COVID-19 era has caught research attention since very first epidemic weeks [37,38,39,40]. The negative correlation between smoking and humoral response to vaccines has been widely described [41,42], and the lower occurrence of AEFI in smokers suggests a potential ancillary effect of tobacco use on the impairment of adequate innate and adaptive immune responses that underpin AEFI occurrence and, as is likely, general COVID-19 vaccine responses, which also reflect the change in smokers’ risk of breakthrough infection compared with the same risk in the pre-vaccination era [8,39].

Compared with participants receiving the BNT162b2 vaccine, those receiving mRNA-1273 had a higher risk of reporting tiredness/fatigue and joint pain, and the risk was even tripled with nausea. The finding of increased AEFI in mRNA-1273 recipients has been widely observed worldwide, and it is consistent with reactions reported following mRNA-1273 primary series and booster (also irrespectively of potential mix-and-match of doses) [19,43,44,45]. However, the mechanisms of differences in safety profiles of the two mRNA-based platforms deserve further research [44]: of note, mRNA-1273 contains a higher dosage of mRNA (100 µg and 50µg, respectively, for primary and booster doses, vs. 30 µg of BNT162b2) and different lipid nanoparticle components and excipients [46,47]. Some research suggested an additional immunogenicity of the mRNA-1273 vaccine compared to BNT162b2 [19], with higher concentrations of RBD- and N-terminal domain-specific IgA, increased antibodies eliciting neutrophil phagocytosis, and natural killer cell activation [48]. However, no difference in incidence rate of breakthrough infections according to vaccine platforms was described in previous analyses of the MOSAICO cohort [8].

Extending the analysis to investigate the health impact of post-booster dose AEFI (as a proxy of symptom severity), we found that around 20% of the total sample reported the inability to perform normal daily activities, the inability to work, or having sought medical care. Being a woman and having an autoimmune condition significantly increased the odds of suspending daily activities and work, likely due to a higher severity of postvaccination symptoms in these groups, irrespectively of the found risk of repeated AEFI. Instead, HCWs had lower odds of suspending work, as a conceivable consequence of the frontline clinicians shortage that is facing the Italian healthcare industry and system, particularity after the COVID-19 emergency [49]. Likewise, among participants receiving mRNA-1273, the need for medical care was reported more frequently (18.6% vs. 7.0% of BNT162b2 recipients, *p*-value = 0.03), with 2.6-fold statistically significant odds.

In brief, this observational study described AEFI occurrence after post-booster dose of the mRNA COVID-19 vaccine, mostly describing mild and short-lived symptoms which represent common signals of stronger immune reaction and adequate vaccine response. In addition, when interviewed about post-booster AEFI, participants did not mention any late onset of AEFI after previous vaccinations. These findings therefore confirm the global safety profile of COVID-19 vaccines, with an overall benefit of vaccination that outweighs the risks of side effects. In this sense, real-world evidence described only rare severe AEFI attributable to mRNA COVID-19 vaccines, as for any vaccination [9,10]. The last iteration of the COVID-19 Vaccine Surveillance Report steered by the Italian Medicines Agency updated the list of mRNA vaccines’ adverse events of interest, which includes cases of anaphylaxis (~3 cases per 1,000,000 vaccine doses administered), pericarditis (~5,5 cases per 1,000,000 vaccine doses) and myocarditis (up to 10 cases per 1,000,000 vaccine doses in male aged 12 to 29 years) [50].

This paper has a number of strengths and weaknesses. The study offers an interesting insight into the risk of postvaccination AEFI based on individual predisposing factors. Moreover, the cohort was carefully assembled, being representative of the population aimed to study and thus providing reliable estimates of the research outcomes. Despite these strengths, some limitations should be acknowledged. First, the number of participant HCWs who were included in the analysis may be limited as a real-world study, if compared with other surveys on COVID-19 vaccine safety. However, the sample size was satisfactory, and AEFI rate in line with existing similar research [14,20,22]. In this sense, it is also worth noting that other findings from the MOSAICO publication [8] were confirmed by the emerging literature [51,52]. Second, being a working-age population, our cohort did not allow us to investigate the occurrence and predictors of AEFI in other vulnerable populations, including older persons and those with comorbidities, as well as children and adolescents. Third, due to its design, the study did not consider all possible individual predisposing factors and bias which may have had an effect on AEFI onset and reporting. However, we included those mainly associated with the outcomes, and the use of robust statistical analysis allowed us to select the explanatory factors potentially correlated with such confounders. Fourth, although the reported AEFI are commonly associated with COVID-19 vaccination, it is not possible to indicate to what extent a possible notoriety bias—defined as “a selection bias in which a case has a greater chance of being reported if the subject is exposed to the studied factor known to cause, thought to cause, or likely to cause the event of interest” [53]—may have affected AEFI over-reporting and, therefore, risk prediction. We included the participants’ professional role (HCWs vs. non-HCWs) as an explanatory variable to soften this potential bias attributable to specialized knowledge. Lastly, we were not able to measure the duration, persistence, and severity of the symptoms; however, the use of proxies of AEFI’s health impact (i.e., inability to perform normally and to work, and seeking medical care) serves in place of these characteristics.

## 5. Conclusions

In conclusion, this study describes risk predictors for repeated adverse effects following a booster dose of the mRNA COVID-19 vaccine, and the results are useful to update the information about COVID-19 vaccine safety. As a whole, the research offers at least three implications for public health practice: (i) it supports reframing post-booster AEFI symptoms as signals of stronger immune reaction and, therefore, adequate vaccine response against COVID-19; (ii) it provides actionable metrics to inform guidelines for future COVID-19 immunization boosters in HCWs and, more in generally, in adults; (iii) it could be exploited to convey reliable safety information to reassure the wider public in favor of COVID-19 vaccines which are key crucial to control the pandemic burden. In this sense, continuous standard monitoring and long-term follow-ups are required to help maximize the current global vaccination campaign against COVID-19.

## Figures and Tables

**Table 1:** Study population.

	N (%)
Total third-dose recipients	310
Age (in years) *	38 (32–50)
Gender Men Women	127 (41.0) 183 (59.0)
Role HCWs Non-HCWs	239 (77.1) 71 (22.9)
Cigarette smoker Never/Former Current	195 (62.9) 115 (37.1)
Health status Previous SARS-CoV-2 infection Autoimmune disease Immunosuppressive therapy *^§^*	50 (16.1) 10 (3.2) 5 (1.6)
mRNA vaccine platform received BNT162b2 mRNA-1273	259 (83.5) 51 (16.5)
Time interval between primary vaccination cycle and booster dose (in weeks) * ^	36 (35–42)
Reporting at least one AEFI	258 (83.2)

* Summarized by median and interquartile range (IQR). § Computed on 309 individuals; *^^^* Computed on 301 individuals. Abbreviations: HCWs, healthcare workers; SARS-CoV-2, Severe Acute Respiratory Syndrome Coronavirus 2; AEFI, adverse effects following immunization.

**Table 2:** Risk ratios (RR) and 95% confidence intervals (95%CI) indicating associations between the occurrence of adverse effects following immunization (AEFI) and characteristics evaluated (N = 310).

Risk Ratio (RR) and 95% Confidence Interval (95%CI)												
AEFI	Cumulative Incidence N (%)	Same AEFI after the First Dose	Same AEFI after the Second Dose	Age (in Years)	Gender (Women)	Previous SARS-CoV-2 Infection	Professional Role (HCWs)	Smokers	Autoimmune Disease	Immunosuppressive Therapy	Vaccine Platform (mRNA-1273)	Time since Primary Vaccination Cycle (in Weeks)
Pain at injection site	228 (73.6)	3.97 (2.09–7.54) *	1.90 (1.16–3.09) **		1.12 (1.00–1.25)	**1.17 (1.01–** 1.34) ***			1.11 (0.92– 1.34)	1.15 (0.85– 1.57)		1.01 (1.00– 1.01)
Redness at injection site	54 (17.4)	15.36 (8.97–26.30) *			1.21 (0.79–1.84)		1.34 (0.84–2.14)		0.59 (0.25– 1.38)	§		**0.97 (0.94–** 0.997) ***
Swelling at injection site	66 (21.3)	7.19 (3.17–16.34) *	1.92 (0.91–4.06)			1.27 (0.81– 1.98)						1.02 (1.00– 1.04)
Itch at injection site	20 (6.5)	3.23 (0.79–13.27)	9.95 (2.01–49.42) **						3.94 (0.85–18.30)	§	0.18 (0.02–2.00)	1.08 (0.96– 1.21)
Tiredness and fatigue	146 (47.1)	3.56 (2.33–5.44) *	1.56 (1.12–2.16) **		1.31 (1.04–1.65) ***	0.83 (0.65– 1.06)	0.87 (0.72–1.05)			2.01 (0.88– 4.57)	1.27 (1.02–1.68) ***	1.01 (0.99– 1.02)
Headache	118 (38.1)	3.75 (2.23–6.33) *	1.91 (1.31–2.80) *		1.45 (1.08–1.94) **			0.85 (0.67–1.07)		0.36 (0.08– 1-75)	1.18 (0.92–1.50)	
Muscle pain	134 (43.2)	6.25 (3.60–10.88) *	1.30 (1.00–1.69)		1.23 (0.98–1.55)			0.79 (0.64–0.96) ***	0.69 (0.37– 1.28)		1.19 (0.97–1.47)	
Joint pain	112 (36.1)	5.77 (3.24–10.25) *	1.54 (1.08–2.18) ***	1.02 (1.01–1.03) **	1.22 (0.93–1.59)	0.82 (0.57– 1.17)		0.86 (0.68–1.09)	0.75 (0.42– 1.38)		1.43 (1.11–1.84) **	
Chills	90 (29.0)	7.64 (4.25–13.73) *	1.49 (1.06–2.09) ***	1.02 (1.01–1.03) **	1.31 (0.93–1.86)	0.75 (0.50– 1.12)			0.68 (0.30– 1.52)		1.35 (0.97–1.87)	
Fever	67 (21.6)	6.21 (3.69–10.44) *	1.51 (1.05–2.14) ***		1.35 (0.91–2.01)			0.61 (0.41–0.94) ***				1.01 (0.99– 1.03)
Swelling of lymph nodes	35 (11.3)	5.39 (2.18–13.36) *	1.76 (0.69–4.45)			1.55 (0.65– 3.68)	1.66 (0.68–4.05)		§	§		
Abdominal pain	18 (5.8)	4.86 (1.19–19.77) ***	5.31 (1.31–21.47) ***			1.74 (0.59– 5-12)	0.61 (0.26–1.44)		§	§		
Nausea	26 (8.4)	16.68 (4.96–56.06) *	2.65 (1.03–6.82) ***	1.01 (0.99–1.04)	1.94(0.59–6.29)			0.38 (0.19–0.77) **			3.07 (1.30–7.25) **	
Vomiting ^	5 (1.6)											
Shortness of breath ^	7 (2.3)											
Rash ^	4 (1.3)											
Swelling of face, tongue, or throat^	2 (0.7)											
Diarrhea ^	1 (0.3)											
Sleep disturbances ^	1 (0.3)											
Dizziness ^	1 (0.3)											
Reactivation of Herpes Zoster Virus ^	1 (0.3)											

Statistically significant RRs (and 95%CIs) are in bold: * p-value ≤ 0.001; ** p-value ≤ 0.01; *** p-value ≤ 0.05; Empty cells indicate that covariates were omitted for *p*-value > 0.4; § Not included for low prevalence in the sub-group; ^ Not estimated for total cumulative incidence lower than 5%. Abbreviations: HCWs, healthcare workers; AEFI, adverse effects following immunization.

**Table 3:** Health impact of AEFI after third dose of COVID-19 vaccines.

	N (%)
Inability to perform normal daily activities	57 (18.4)
Inability to work	59 (19.0)
Seeking medical care	25 (8.1)
Remote medical consultation	21 (6.8)
Medical examination	5 (1.6)
Remote consultation with vaccination center	1 (0.3)
Medical examination at vaccination center	1 (0.3)
Emergency department or hospital access	0 (0)

**Table 4:** Multivariate logistic regression models predicting AEFI health impact.

Model 1: Odds of Inability to Perform Normal Daily Activities Due to AEFI				
Variable	OR	SE	95% CI	*p*-Value
Log likelihood = − 137.63; *χ*^2^ = 20.21 (5 df); *p*-value = 0.001				
*Gender* Men Women	*Ref.* 2.45	- 0.85	- 1.23–4.85	0.01
*Role* Non-HCW HCWs	*Ref.* 0.57	- 0.21	- 0.28–1.17	0.13
*Autoimmune disease* No Yes	*Ref.* 11.71	- 10.55	- 2.00–68.48	0.006
*Immunosuppressive therapy* No Yes	*Ref.* 0.23	- 0.32	- 0.02–3.45	0.29
*Type of booster vaccine* BNT162b2 mRNA-1273	*Ref.* 1.95	- 0.72	- 0.95–4.03	0.07
Model 2: Odds of inability to work due to AEFI				
Variable	OR	SE	95% CI	*p*-value
Log likelihood = − 144.53; *χ*^2^ = 12.28 (4 df); *p*-value = 0.02				
*Gender* Men Women	*Ref.* 2.04	- 0.68	- 1.07–3.91	0.03
*Role* Non-HCW HCWs	*Ref.* 0.48	- 0.16	- 0.24–0.94	0.03
*Autoimmune disease* No Yes	*Ref.* 4.10	- 2.90	- 1.03–16.37	0.05
*Type of booster vaccine* BNT162b2 mRNA-1273	*Ref.* 1.45	- 0.54	- 0.70–3.02	0.32
Model 3: Odds of seeking medical care due to AEFI				
Variable	OR	SE	95% CI	*p*-value
Log likelihood = − 80.74; *χ*^2^ = 12.17 (5 df); *p*-value = 0.03				
*Gender* Men Women	*Ref.* 2.36	- 1.17	- 0.89–6.25	0.08
*Age* (continuous, in years)	1.03	0.2	0.99–1.07	0.14
*Role* Non-HCW HCWs	*Ref.* 0.41	- 0.19	- 0.16–1.02	0.06
*Immunosuppressive therapy* No Yes	*Ref.* 2.79	- 3.34	- 0.27–29.02	0.39
*Type of booster vaccine* BNT162b2 mRNA-1273	*Ref.* 2.61	- 1.25	- 1.03–6.67	0.04

Abbreviations: AEFI, adverse effects following immunization; HCW, healthcare worker; Ref., reference category; OR, odds ratio; 95%CI, 95% confidence interval; SE, standard error; df, degrees of freedom.

## Data Availability

Data and supporting materials associated with this study will be provided upon request by contacting the corresponding author.

## Appendix A

This appendix details the cumulative incidence of AEFI after each of the three doses of the COVID-19 vaccination series.

**Table A1:** Cumulative incidence of adverse effects following each dose of the COVID-19 vaccination series.

Adverse Effect	After First Dose (N = 320)	After Second Dose (N = 308)	After Booster Dose (N = 310)
	N (%)	N (%)	N (%)
Pain at injection site	266 (83.1)	250 (81.2)	228 (73.6)
Redness at injection site	58 (18.1)	54 (17.5)	54 (17.4)
Swelling at injection site	85 (26.6)	66 (21.4)	66 (21.3)
Itch at injection site	24 (7.5)	20 (6.5)	20 (6.5)
Tiredness and fatigue	162 (50.6)	154 (50.0)	146 (47.1)
Headache	127 (39.8)	140 (45.4)	118 (38.1)
Muscle pain	143 (44.7)	158 (51.3)	134 (43.2)
Joint pain	120 (37.5)	133 (43.2)	112 (36.1)
Chills	99 (30.9)	117 (38.0)	90 (29.0)
Fever	57 (17.8)	86 (27.9)	67 (21.6)
Swelling of lymph nodes	22 (6.9)	21 (6.8)	35 (11.3)
Abdominal pain	14 (4.4)	13 (4.2)	18 (5.8)
Nausea	32 (10.0)	35 (11.4)	26 (8.4)
Vomiting	10 (3.1)	10 (3.2)	5 (1.6)
Shortness of breath	7 (2.2)	5 (1.6)	7 (2.3)
Rash	4 (1.2)	5 (1.6)	4 (1.3)
Swelling of face, tongue, or throat	4 (1.2)	1 (0.3)	2 (0.7)
Diarrhea	1 (0.3)	Not reported	1 (0.3)
Sleep disturbances	1 (0.3)	1 (0.3)	1 (0.3)
Dizziness	1 (0.3)	Not reported	1 (0.3)
Reactivation of herpes zoster virus	1 (0.3)	1 (0.3)	1 (0.3)
Venous phlebitis	1 (0.3)	1 (0.3)	Not reported
Left-sided facial numbness	1 (0.3)	1 (0.3)	Not reported
